# Involvement of FvSet1 in Fumonisin B1 Biosynthesis, Vegetative Growth, Fungal Virulence, and Environmental Stress Responses in *Fusarium verticillioides*

**DOI:** 10.3390/toxins9020043

**Published:** 2017-01-24

**Authors:** Qin Gu, Hafiz Abdul Samad Tahir, Hao Zhang, Hai Huang, Tiantian Ji, Xiao Sun, Liming Wu, Huijun Wu, Xuewen Gao

**Affiliations:** Key Laboratory of Monitoring and Management of Crop Diseases and Pest Insects, Department of Plant Pathology, College of Plant Protection, Nanjing Agricultural University, Ministry of Education, Nanjing 210095, China; guqin@njau.edu.cn (Q.G.); 2014202054@njau.edu.cn (H.A.S.T.); 2014102015@njau.edu.cn (H.Z.); huanghai_201510@163.com (H.H.); 2015102015@njau.edu.cn (T.J.); 2016102012@njau.edu.cn (X.S.); wuliming@njau.edu.cn (L.W.)

**Keywords:** *Fusarium verticillioides*, FvSet1, fumonisin B1, fungal growth and virulence, stress responses

## Abstract

*Fusarium verticillioides* (teleomorph, *Gibberella moniliformis*) is an important plant pathogen that causes seedling blight, stalk rot, and ear rot in maize (*Zea mays*). During infection, *F. verticillioides* produce fumonsins B1 (FB1) that pose a serious threat to human and animal health. Recent studies showed that Set1, a methyltransferase of H3K4, was responsible for toxin biosynthesis in filamentous fungi. However, to date, the regulation of FvSet1 on FB1 biosynthesis remains unclear. In the current study, we identified only one Set1 ortholog in *F. verticillioides* (FvSet1) and found that the deletion of *FvSET1* led to various defects in fungal growth and pathogenicity. More interestingly, the *FvSET1* deletion mutant (ΔFvSet1) showed a significant defect in FB1 biosynthesis and lower expression levels of *FUM* genes. FvSet1 was also found to play an important role in the responses of *F. verticillioides* to multiple environmental stresses via regulating the phosphorylation of FvMgv1 and FvHog1. Taken together, these results indicate that FvSet1 plays essential roles in the regulation of FB1 biosynthesis, fungal growth and virulence, as well as various stress responses in *F. verticillioides.*

## 1. Introduction

*Fusarium verticillioides* (teleomorph, *Gibberella moniliformis*) is an important plant pathogen that causes seedling blight, stalk rot, and ear rot in maize (*Zea mays*), which is one of the most common diseases of affecting maize worldwide [1,2]. More importantly, during growth in maize, *F. verticillioides* produces various mycotoxins, such as fumonisins, which are extremely harmful to human and animal health [3,4]. Fumonisins have been shown to play critical roles in the development of maize diseases caused by *F. verticillioides* [5]. Among the diverse forms of the fumonisins, fumonisins B1 (FB1) is the most prevalent and well-characterized. A cluster of 17 *FUM* genes is known to be responsible for FB1 biosynthesis [6,7]. However, the molecular mechanisms underlying the regulation of *FUM* gene transcription in *F. verticillioides* remains to be elucidated.

In eukaryotic cells, regulation of gene transcription is associated with multiple modifications of histones such as methylation, phosphorylation, adenosine diphosphate (ADP)-ribosylation, biotinylation, acetylation, and ubiquitination [8]. Among these modifications, histone H3 lysine 4 methylation (H3K4me) was found to activate gene transcription in various organisms, including yeast, plants, and animals [9,10,11,12]. Recent studies have shown that methylation of lysine 4 on histone H3 has important influences on secondary metabolism and fungal virulence in several pathogenic fungi, such as *Fusarium graminearum* and *Magnaporthe oryzae*. In *F. graminearum*, H3K4me plays important roles in deoxynivalenol (DON) production by positively regulating transcription of *TRI* genes, which encode trichothecene biosynthetic enzymes and regulators [13]. A global view of the relationship between H3K4me and gene transcription in *M. oryzae* obtained by CHIP-seq analysis revealed an association between H3K4me and activation of gene expression and regulation of fungal virulence [14].

In *Saccharomyces cerevisiae*, the Set1/COMPASS complex, consisting of Set1 and its subunits, functions as a methyltransferase capable of catalyzing the mono-, di-, and trimethylation of H3K4 [15,16,17]. Deletion of *S. cerevisiae* Set1 resulted in transcriptional silencing and growth defects [18]. Similar to *S. cerevisiae*, Set1 is essential for H3K4me in diverse pathogenic fungi, including *F. graminearum* and *M. oryzae*. Disruption of *SET1* also leads to abnormal secondary metabolism and defects in fungal virulence [13,14]. In addition, a recent study in *F. graminearum* revealed that FgSet1 is associated with multiple stress responses that extend our understanding of the functions of H3K4 methylation on environmental stress responses in filamentous fungi [13].

In this study, we identified the *FvSET1*, which encodes H3K4 methyltransferase. For genetic characterization of FvSet1, we generated the *FvSET1* deletion mutant. Phenotypic analysis showed that FvSet1 plays critical roles in FB1 biosynthesis, fungal growth and virulence, and stress responses in *F. verticillioides*. Moreover, in the current study, it was found that FvSet1 is required for activation of *FUM* gene expression. These findings provide a clearer understanding of regulation of FB1 biosynthesis in *F. verticillioides*, which will be beneficial to establishing the efficient strategies for FB1 contamination management.

## 2. Results

### 2.1. In Silico Analysis of FvSet1 in F. verticillioides

To elucidate the function of H3K4me in *F. verticillioides*, we searched for the ortholog of *S. cerevisiae* Set1, which is the core component of methyltransferase complex Set1/COMPASS. Using *S. cerevisiae* Set1 as a query, we found only one Set1 ortholog, FVEG_07811 (designated *FvSET1*), from the *F. verticillioides* genome via BLASTP. *FvSET1* was 3901 bp in length and interrupted with three introns. FvSet1 was predicted to encode a 1234 amino acids protein and harbor two typical SET domains. The putative amino acids sequences of FvSet1 shared 56% identity with that of yeast Set1 (Figure S1A). Additionally, phylogenic analysis showed that the FvSet1 was highly homologous to its counterparts in yeast and other pathogenic fungi (Figure S1B). 

### 2.2. Deletion and Complementation of FvSet1 in F. verticillioides

To characterize the function of FvSet1, we generated a *FvSET1* deletion mutant (ΔFvSet1) using a homologous recombination strategy (Figure S2A). Among 28 transformants, three *FvSET1* deletion mutants were identified by polymerase chain reaction (PCR) analysis with the primer pair A5 + A6 (Table S1). Southern blot analysis confirmed that ΔFvSet1 resulted from the anticipated homologous recombination of *FvSET1* (Figure S2B). To confirm that various defects in ΔFvSet1 were the result of *FvSET1* deletion, a full-length *FvSET1* was transformed into the ΔFvSet1 deletion mutant and the resulting complementation strain ΔFvSet1-C was identified by PCR analysis using the A5 + A6 primer pairs.

### 2.3. FvSet1 Is Involved in Fungal Growth and Conidial Germination of F. verticillioides

**Figure 1:**
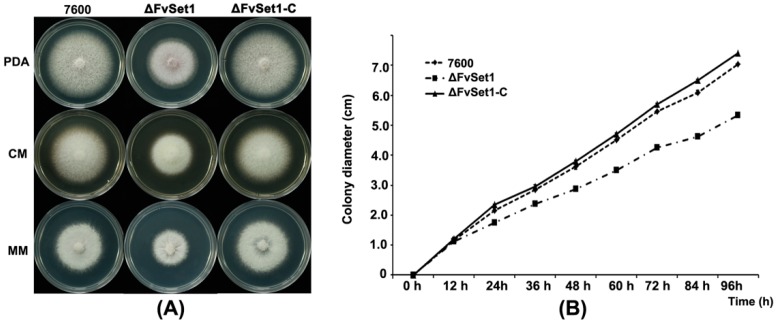
Impacts of *FvSET1* deletion on *F. verticillioides* mycelial growth. (**A**) the wild-type (strain 7600), the *FvSET1* deletion mutant (ΔFvSet1), and the complementation strains (ΔFvSet1-C) were cultured on PDA (Potato Dextrose Agar), CM (Complete Medium) and MM (Minimal Medium) at 25 °C for four days; and (**B**) comparison of mycelial growth rates among the wild-type, ΔFvSet1, and ΔFvSet1-C on PDA medium. Line bars in each column denote standard errors of three repeated experiments.

To characterize the function of FvSet1 in mycelial growth, each strain was cultured at 25 °C on potato dextrose agar (PDA), complete medium (CM), and minimal medium (MM). After four days, ΔFvSet1 displayed a significant decreased growth rate (Figure 1). 

**Figure 2:**
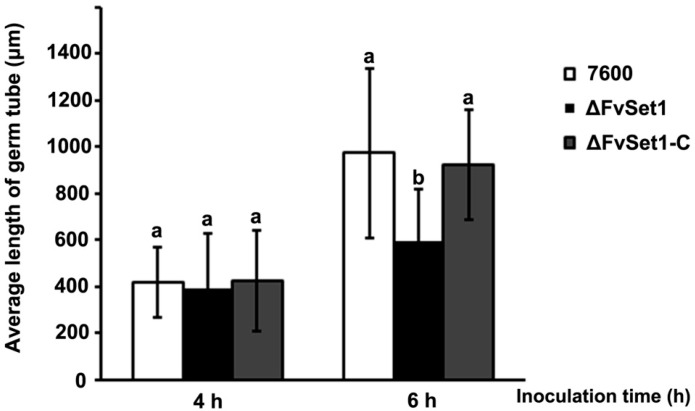
Involvement of FvSet1 in conidial germination in *F. verticillioides*. Average length of 150 germ tubes from each strain after incubation for 4 h and 6 h of in YEPD (Yeast Extract Peptone Dextrose Medium). Bars denote standard errors from three repeated experiments.

To detect the roles of FvSet1 in asexual development, fresh mycelia (50 mg) of each strain were cultured in liquid CMC medium at 25 °C in a shaking incubator. After four days, ΔFvSet1 did not exhibit any significant defects in conidiation (data not shown). To determine the effect of *FvSET1* deletion on conidal germination, conidia from each strain was used to inoculate in YEPD (Yeast Extract Peptone Dextrose) liquid medium and cultured at 25 °C for 4 h and 6 h. It was found that ΔFvSet1 showed a similar percentage of conidial germination compared with that of the wild-type 7600 strain and the ΔFvSet1-C complementation strain (data not shown). However, the average length of germinated conidial germ tubes of the mutant was dramatically reduced when compared with those of the wild-type 7600 after incubation for 4 h and 6 h (Figure 2).

### 2.4. FvSet1 Is Indispensable for Full Virulence in F. verticillioides

**Figure 3:**
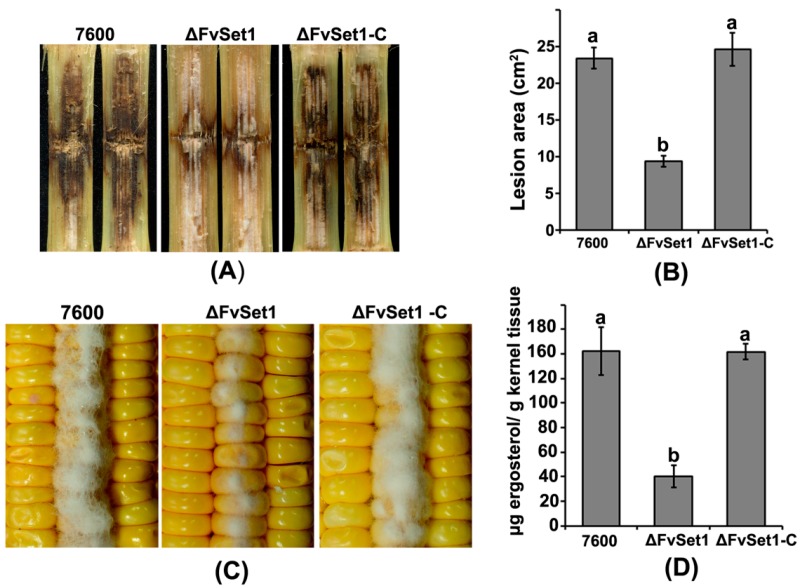
FvSet1 is required for full virulence in *F. verticillioides*. (**A**) maize stalk infection assays. Maize stalks were injected with conidial suspensions of the wild-type (Strain 7600), the *FvSET1* deletion mutant (ΔFvSet1), and the complementation strain (ΔFvSet1-C). Longitudinal dissections of infected maize stalks were photographed 15 days after inoculation; (**B**) lesion area of longitudinally dissected maize stalks infected with each strain for 15 days; (**C**) the wounded maize kernels were inoculated with conidial suspension of wild-type, ΔFvSet1, and ΔFvSet1-C. Infected maize kernels were photographed 15 days after inoculation; and (**D**) growth of the wild-type, ΔFvSet1, and ΔFvSet1-C strains on living maize kernels was evaluated by analysis of ergosterol biosynthesis. Values represent the mean ergosterol concentration in eight maize kernels sampled in triplicate from three separate ears for each strain. Line bars in each column denote standard errors of three repeated experiments.

To test the pathogenicity of each strain, we injected the conidia of the wild-type 7600, ΔFvSet1, and ΔFvSet1-C into maize stalks. After 15 days, the typical symptoms of rot caused by ΔFvSet1 were significantly reduced when compared with the effects of the wild-type 7600 and the ΔFvSet1-C complementation strain on maize stalks under the same condition (Figure 3A,B). To investigate the roles of FvSet1 on colonization, damaged maize kernels were sterilized and then inoculated with a 10-μL aliquot of conidial suspension of each strain. After seven days, a dramatic reduction inaerial hyphae in ΔFvSet1 was observed during pathogenesis compared with those of the wild-type and ΔFvSet1-C (Figure 3C). Furthermore, we examined the amount of fungal ergosterol in the inoculated maize kernels. As indicated in Figure 3D, a four-fold reduction in the amount of ergosterol produced by ΔFvSet1 was observed compared with that produced by wild-type and ΔFvSet1-C. These results strongly suggested that FvSet1 plays a critical role in virulence of *F. verticillioides* on maize.

### 2.5. FvSet1 Is Required for FB1 Biosynthesis in F. verticillioides

**Figure 4:**
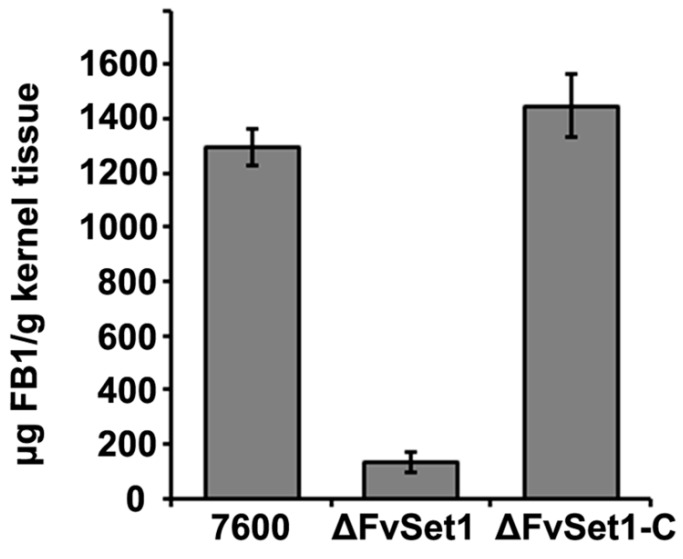
Effects of *FvSET1* deletion on FB1 biosynthesis in *F. verticillioides*. The amounts of FB1 produced by each strain in infected maize kernels 21 days after inoculation. Line bars in each column denote standard errors of three replicated experiments.

To determine the function of FvSet1 in FB1 biosynthesis, we analyzed the FB1 production in cracked maize kernels inoculated by wild-type, ΔFvSet1, and ΔFvSet1-C. At 21 days post-inoculation, the amounts of FB1 produced by the wild-type and complementation strains were 10-fold higher than that produced by ΔFvSet1 (Figure 4). Additionally, the *FUM* gene expression levels in ΔFvSet1 were significantly reduced compared with that in the wild-type following inoculation of liquid GYAM (Figure S3). These results showed that FvSet1 plays an important role in FB1 production by *F. verticillioides* via regulation of *FUM* gene transcription.

### 2.6. FvSet1 Is Involved in Responses to Multiple Stresses Response

**Figure 5:**
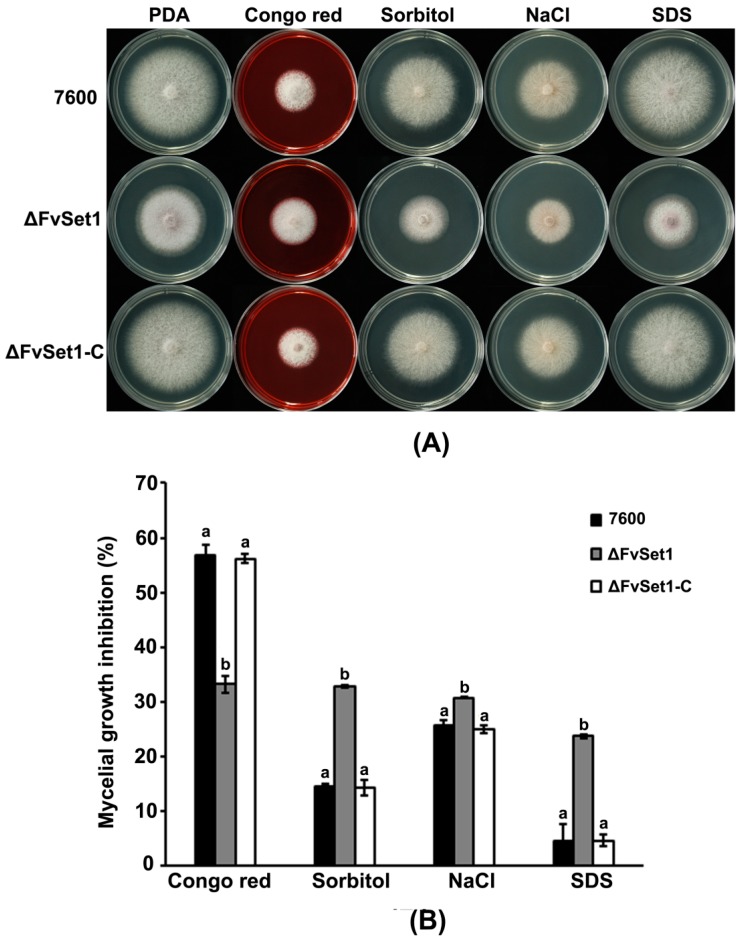
Sensitivity of the wild-type strain of *F. verticillioides* (Strain 7600), the *FvSET1* deletion mutant (ΔFvSet1), and the complementation strain (ΔFvSet1-C) to osmotic and oxidative stresses, and cell wall-damaging agents. (**A**) Comparisons were made on PDA (Potato Dextrose Agar) with or without 0.02% (*w*/*v*) congo red, 1 M sorbitol, 0.7 M NaCl, and 0.02% (*w*/*v*) SDS (Sodium dodecyl sulfate); and (**B**) mycelial growth inhibition was examined after each strain was incubated for three days on PDA supplemented with each compound. Bars denote standard errors from three experiments.

**Figure 6:**
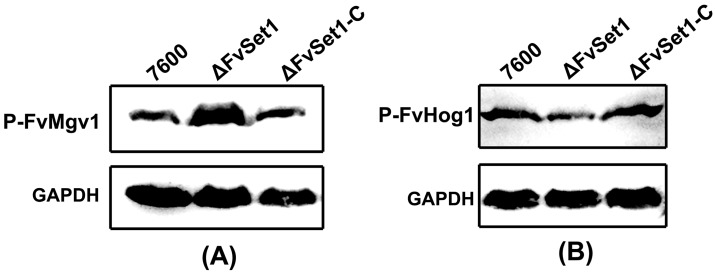
Involvement of FvSet1 in FvMgv1 and FvHog1 phosphorylation. Comparisons of phosphorylated FvMgv1 (**A**) and FvHog1 (**B**) in the wild-type of *F. verticillioides* (strain 7600), the FvSET1 deletion mutant (ΔFvSet1) and the complementation strain (ΔFvSet1-C). Phosphorylated FvMgv1 (P-FvMgv1) and FvHog1 (P-FvHog1) proteins were detected using the phospho-p44/42 and phospho-p38 MAP kinase antibody, respectively.

To characterize the functions of FvSet1 in multiple stress responses, we tested the sensitivity of wild-type, ΔFvSet1, and ΔFvSet1-C to various environmental stresses. As indicated in Figure 5, ΔFvSet1 displayed significant increased sensitivity to osmotic stresses (0.7 M NaCl or 1.0 M sorbitol) and oxidative stress [0.02% SDS (Sodium dodecyl sulfate)] compared with the wild-type and the complementation strain. However, ΔFvSet1 exhibited dramatically increased resistance to a cell wall-damaging agent (0.02% Congo red) compared with that of the wild-type and ΔFvSet1-C (Figure 5). In *S. cerevisiae*, the mitogen-activated protein kinase Mpk1 and Hog1 phosphorylation are required for maintenance of cell wall integrity and response to osmotic stresses, respectively. Thus, in this study, we also analyzed phosphorylation of FvMgv1 (an ortholog of *S. cerevisiae* Mpk1) and FvHog1 (an ortholog of *S. cerevisiae* Hog1) in *F. verticillioides*. As indicated in Figure 6A, the level of FvMgv1 phosphorylation in ΔFvSet1 was dramatically higher than that in the wild-type and the complementation strain ΔFvSet1-C. However, a decreased level of FvHog1 phosphorylation was observed in ΔFvSet1 when compared with that in the wild-type and ΔFvSet1-C (Figure 6B). These results indicated that FvSet1 play an important role in response of multiple environmental stresses by regulating the phosphorylation of FvMgv1 and FvHog1.

## 3. Discussion

Methylation of H3K4 is generally associated with transcriptional activation in eukaryotic cells [19,20]. Set1, the core component of methyltransferase Set1/COMPASS, was responsible for catalyzing the mono-, di-, and trimethylation of H3K4, which is highly conserved from yeast to humans [21]. Recently, Set1 was reported to play important roles in fungal growth, virulence, and secondary metabolism in several pathogenic fungi. However, the contribution of Set1 in *F. verticillioides* remains to be defined. Thus, in this study, we investigated the roles of FvSet1 on mycotoxin biosynthesis, fungal virulence, and fungal growth in *F. verticillioides*.

Recent studies show that the expression of the secondary metabolite cluster is associated with H3K4me [22]. In *Aspergillus nidulans*, CclA, which is a member of the Set1/COMPASS complex, plays important roles in di- and trimethylation of H3K4. The *CCLA* deletion mutant showed increased production of several secondary metabolites [23]. In contrast to its roles as a negative regulator of secondary metabosim in *A. nidulans*, FgSet1 positively regulates secondary metabolism in *F. graminearum*. Deletion of *FgSET1* is associated with dramatic defects in DON and aurofusarin biosynthesis. In *F. verticillioides*, the biochemical pathway for fumonisin biosynthesis is well established. The *FUM* gene cluster consists of 17 *FUM* genes at a single locus and is responsible for encoding FB1 biosynthetic enzymes and regulators [6]. However, to date, the regulation of *FUM* gene cluster is relatively unclear. In this study, we found a dramatic reduction of FB1 production in ΔFvSet1 compared with that in the wild-type and complementation strain, which is in accordance with what is known in *F. graminearum*. (Figure 4). Furthermore, *FUM* gene expression decreased significantly in ΔFvSet1, indicating that FvSet1 positively regulates FB1 biosynthesis at the level of *FUM* gene transcription (Figure S3). Taken together, these results showed that functions of H3K4me in regulation of secondary metabolism vary significantly in different eukaryotes. 

In *S. cerevisiae*, Set1 null mutants exhibited morphological, developmental and growth defects [24]. Similarly, FvSet1 was shown to be associated with fungal growth and virulence in *F. graminearum* and *M. oryzae* [13,14]. In the current study, *FvSET1* deletion mutants displayed similar defects in fungal growth and conidial germination (Figure 1 and Figure 2). More importantly, pathogenicity tests showed that ΔFvSet1 exhibited significantly reduced virulence in maize stalks and kernels, suggesting that FvSet1 is essential for fungal virulence in *F. verticillioides* (Figure 3). These results suggest that FvSet1 play similar roles in vegetative growth and the pathogenesis of pathogenic fungi.

To date, information regarding the relationship between H3K4me and environmental stress responses is scarce. Thus, in this study, we conducted sensitivity assays of various environmental stresses including osmotic, oxidative, and cell wall stresses. *FvSET1* deletion led to increased sensitivity to osmotic stresses (NaCl or sorbitol) and oxidative stress (SDS), while resistance to the cell wall-damaging agent (congo red) was significantly increased (Figure 5). Consistent with this finding, ΔFvSet1 showed a higher level of FvMgv1 phosphorylation and a decreased level of FvHog1 phosphorylation in comparison with those in the wild-type and the complementation strain (Figure 6). However, in *F. graminearum*, ΔFgSet1 did not exhibit a detectable change in sensitivity to osmotic and oxidative stresses [13]. Taken together, these results indicated that FvSet1 play a distinct role in response to multiple environmental stresses in *F. verticillioides*.

## 4. Materials and Methods

### 4.1. Fungal Strains, Growth Conditions and Sporulation Tests

For the wild-type strain of *F. verticillioides* strain 7600, the deletion mutants derived from the wild-type and the complementation strains used in our study were cultured at 25 °C on complete medium (CM) agar (10 g glucose, 2 g peptone, 1 g yeast extract, 1 g casamino acids, nitrate salts, trace elements, 0.01% vitamins, 10 g agar and 1 L water, pH 6.5) [25], potato dextrose agar (PDA) (200 g potato, 20 g glucose, 10 g agar and 1 L water), and minimal medium (MM) agar (10 mM K_2_HPO_4_, 10 mM KH_2_PO_4_, 4 mM (NH_4_)_2_SO_4_, 2.5 mM NaCl, 2 mM MgSO_4_, 0.45 mM CaCl_2_, 9 mM FeSO_4_, 10 mM glucose, 1% agar, pH 6.9) for mycelial growth tests.

To characterize the conidiation of each strain, we added 50 mg mycelia into a 50-mL flask containing 20 mL liquid CMC medium (1 g NH_4_NO_3_, 1 g KH_2_PO_3_, 0.5 g MgSO_4_·7H_2_O, 1 g yeast extract, 15 g CMC and 1 L water). The flasks were incubated at 25 °C for 4 days in a shaker (180 rpm). Conidia were counted by using a hemacytometer (Baili, Shanghai, China). The experiment was repeated on three independent occasions. 

### 4.2. Constructed Gene Deletion and Complemented Strains

The *FvSET1* replacement construct was generated using the double-joint PCR approach. The constructs were transformed into the protoplasts of *F. verticillioides* using the protocols described in previous described protocols [26]. The primers used to amplify the sequences upstream and downstream of *FvSET1* are listed in Table S1. Putative mutants were identified by PCR using the relevant primers (Table S1), and were confirmed by Southern blotting assay (Figure 2B). All the strains used in this study were preserved in 30% glycerol at −70 °C.

### 4.3. Pathogenicity Assays

To test the pathogenicity of wild-type, deletion mutant (ΔFvSet1), and the complementation (ΔFvSet1-C) strain on maize stalks, conidia of each strain were collected by filtration through three layers of cotton gauze and subsequently re-suspended in sterilized water at 10^6^ conidia/mL. A sample of the conidial suspension (10 μL) was used to inoculate maize cultivar B73 via a hole punched in the stem. The control maize stalks were inoculated with 10-μL of sterilized water. Six replicates were prepared for each strain. After inoculation, the maize stalks were maintained at 25 ± 2 °C under 80% humidity for 15 days. Fifteen days after inoculation, the infected maize plants were recorded. The experiment was repeated on four independent occasions.

To examine ability of each strain to infect maize kernels, a 10-μL aliquot of a conidial suspension was used to inoculate the damaged maize kernels after surface sterilization. Five replicates were prepared for each strain. Maize kernels were incubated at 25 °C under 100% humidity with a 12 h of daylight period, and were photographed 7 days after inoculation. The experiment was repeated on three independent occasions.

### 4.4. Determination of FB1 Production

A 25-g aliquot of healthy maize kernels was sterilized and inoculated with five mycelial plugs of each strain. After incubation at 25 °C for 21 days, fumonisin B1 was assayed as described previously [27,28]. The amount of FB1 andergosterol in each sample was determined using a HPLC system Waters 1525 (Waters, Milford, MA, USA) [29]. The experiment was repeated three times, and data were analyzed by analysis of variance (ANOVA; SAS version 8.0; SAS Institute, Cary, NC, USA).

### 4.5. RNA Extraction and Quantitative Real-Time PCR

For total RNA extraction, mycelia of each strain were used to inoculate liquid GYAM (0.24 M glucose, 0.05% yeast extract, 8 mM l-asparagine, 5 mM malic acid, 1.7 mM NaCl, 4.4 mM K_2_HPO_4_, 2 mM MgSO_4_, and 8.8 mM CaCl_2_, pH 3.0) or liquid CM for 36 h at 25 °C in the shaking incubator (200 rpm). Mycelia were harvested by filtration over two layers of miracloth and washed with sterilized water. Harvested mycelia were then lyophilized and ground in liquid nitrogen. Total RNA was extracted from mycelia of each sample using the RNAiso Reagent (TaKaRa Co., Dalian, China), and 10 mg of each RNA sample was used for reverse transcription with RevertAid H Minus First Strand cDNA Synthesis Kit employing the oligo(dT)_18_ primer (Fermentas Life Sciences, Burlington, ON, Canada). The expression of each gene was determined by quantitative real-time PCR with the primers listed in Table S1. For each sample, amplification of the actin gene was performed by PCR with the FvActin-F + FvActin-R primer pair (Table S1) was performed as a reference. The experiment was repeated on three independent occasions.

### 4.6. Western Blotting Assay

Six mycelial plugs were inoculated into 150 mL liquid CM and incubated at 25 °C in a shaker (200 rpm) for 36 h. Mycelia were harvested, washed with deionized water and ground in liquid nitrogen. Approximately, 200 mg of finely ground mycelia were re-suspended in 1 mL of extraction buffer [50 mM Tris-HCl, pH 7.5, 100 mM NaCl, 5 mM EDTA (Ethylene Diamine Tetraacetic Acid), 1% Triton X-100, 2 mM PMSF (Phenylmethanesulfonyl fluoride)] and 10-μL of protease inhibitor cocktail (Sangon, Shanghai, China). After homogenization with a vortex shaker, the lysate was centrifuged at 14,000× *g* in a microcentrifuge for 20 min at 4 °C [30]. The resulting proteins were separated by SDS polyacrylamide gel electrophoresis (10% denaturating gel) and transferred to an Immobilon-P transfer membrane (Millipore, Billerica, MA, USA) using electroblotting apparatus (Bio-Rad, Hercules, CA, USA). Phosphor-p44/42 MAP kinase antibody and Phosphor-p38 MAP kinase antibody (Cell Signaling Technology, Boston, MA, USA) were used for detection of phosphorylated FvMgv1 and FvHog1, respectively [13]. GAPDH (glyceraldehyde-3-phosphate dehydrogenase) was detected in samples using anti-GAPDH antibody (Huabio, Hangzhou, China) as a reference. The experiment was repeated on two independent occasions.

## 5. Conclusions

In summary, we genetically and biochemically characterized the functions of FvSet1 in *F. verticillioides*. The results of the current study demonstrate that FvSet1 plays an important role in FB1 biosynthesis, fungal growth and virulence, and environmental stress responses in *F. verticillioides*. These results present a relationship between FvSet1 and FB1 biosynthesis in *F. verticillioides*. However, investigations are still required for elucidating the mechanisms by which H3K4me regulates secondary metabolism.

## Supplementary Materials

The following are available online at www.mdpi.com/2072-6651/9/2/43/s1. Table S1: Primers used in this study. Figure S1: FvSet1 is homologous to those counterparts from yeasts and other filamentous fungi. (**A**) alignments of amino acid sequences of Set1 orthologs from *Fusarium verticillioides* (FvSet1), *Saccharomyces cerevisiae* (ScSet1), *Schizosaccharomyces pombe* (SpSet1), *Neurospora crassa* (NcSet1), *Podospora anserina* (PaSet1), *Colletotrichum gloeosporioides* (CgSet1), *Aspergillus nidulans* (AnSet1), and *Fusarium graminearum* (FgSet1). Boxshade program was used to highlight identical (black shading) or similar (grey shading) amino acids; (**B**) phylogenetic tree generated using the neighbour-joining method with Mega 4.1 software on the basis of the deduced amino acid sequences of FvSet1 from *F. verticillioides* strain 7600 and those from *S. cerevisiae*, *S. pombe*, *N. crassa*, *C. gloeosporioides*, *P. anserine*, *A. nidulans*, and *F. graminearum*. The bootstrap values are indicated on the phylogenetic tree. Figure S2: Schematic representation of the *FvSET1* disruption strategy and Southern blotting analyses of the deletion mutants. (**A**) Schematic diagram of the *FvSET1* gene, and gene replacement construct; (**B**) Southern blot analysis of *Dra* I-digested genomic DNA of the wild type 7600 and ΔFvSet1 mutant hybridized with the *FvSET1* gene and HPH probes, respectively. Figure S3: Relative expression of *FUM* genes in the ΔFvSet1. The relative expression levels of *FUM* genes in ΔFvSet1 are the relative amounts of mRNA of the gene in the wild-type progenitor. The expression level of the actin gene was used as an internal reference for each sample. Line bars in each column denote standard errors of three repeated experiments.
