# Supplementary material for: Involvement of FvSet1 in Fumonisin B1 Biosynthesis, Vegetative Growth, Fungal Virulence, and Environmental Stress Responses in Fusarium verticillioides
Source: Toxins (Basel). 2017 Jan 24;9(2):43. doi: 10.3390/toxins9020043 (PMC5331423; doi:10.3390/toxins9020043)
Supplement: Supplementary file 1 [file toxins-09-00043-s001.pdf]

Supplementary Materials: Involvement of FvSet1 in Fumonisin B1 Biosynthesis, Vegatative Growth, Fungal Virulence, and Environmental Stress Responses in *Fusarium verticillioides*

Qin Gu, Hafiz Abdul Samad Tahir, Hao Zhang, Hai Huang, Tiantian Ji, Xiao Sun, Liming Wu, Huijun Wu and Xuewen Gao

Table S1. Primers used in this study.

Primer	Sequence (5′-3′)	Application
A1	CGATGAAGAGGAGGAGTTTGA	A pair of PCR primers for amplification of the upstream sequence of the *FgSET1* gene for construction of the gene deletion vector
A2	CAAAATAGGCATTGATGTGTTGACCTCCG<br>GAAATTGGTGGGAGAATGA	
A3	CTCGTCCGAGGGCAAAGGAATAGAGTAG<br>AATGATTAGGACGATGACGCC	A pair of PCR primers for amplification of the downstream sequence of the *FgSET1* gene for construction of the gene deletion vector
A4	ATATCGGGGCTCGGGATTT	
A5	AAGCGAGCGTCAACTTCCTTG	A pair of PCR primers for identification of *FgSET1* deletion mutants
A6	ATTGATATAACCGAGGCGACC	
FvSet1-C-F	CAGATCTTGGCTTTCGTAGGAACCCAATC<br>TTCAATGACTCGCCCGCCA	A pair of PCR primers to amplify *FgSET1* fragment used for construction of the FgSet1-C vector
FvSet1-C-R	CACCACCCCGGTGAACAGCTCCTCGCCCT<br>TGCTCAC GTTGAGGAAGCCCTTGCAGTT	
HPH-F	GGAGGTCAACACATCAATGCCTATT	Amplify *HPH* sequence
HPH-R	CTACTCTATTCCTTTGCCCT	
FvActin-F	TGCTCCTGAGGCTCTCTTCCA	Quantitative real-time PCR primers for analysis of the reference gene actin expression level
FvActin-R	AAGCAAGAATAGAACCACCGA	
FUM1-F	TGCTGCCCTGTATCACAACCA	Quantitative real-time PCR primers for analysis of *FUM1* gene expression level
FUM1-R	AATGTGCGCTTGATCCAGTT	
FUM6-F	TCTCTTGTTCTTTGGCTGTCG	Quantitative real-time PCR primers for analysis of *FUM6* expression level
FUM6-R	TCAATTTCTAGCAGCATCGG	
FUM7-F	GCATGGAGAGACAAGTTGCA	Quantitative real-time PCR primers for analysis of *FUM7* expression level
FUM7-R	TCTGATGAAACTGGGCTTCGT	
FUM21-F	AGCTTGTCAACCCAGCAGATA	Quantitative real-time PCR primers for analysis of *FUM21* expression level
FUM21-R	TCAACTATAGTCCCGGCATCA	
FUM10-F	TTTGGAACCCAATGGCGAT	Quantitative real-time PCR primers for analysis of *FUM10* expression level
FUM10-R	TTTCGGCAGGGCTGATTTTT	
FUM11-F	AAGGGGGGAAGATAGGCACT	Quantitative real-time PCR primers for analysis of *FUM11* expression level
FUM11-R	ATTACGAGTCTTAGCGAGCGA	
FUM13-F	AAACCATGGGATGGTATCAGG	Quantitative real-time PCR primers for analysis of *FUM13* expression level
FUM13-R	TTTCTGCTGAGCCGACATCAT	
FUM14-F	AAGAGGTGCTAAAGACAGCCA	Quantitative real-time PCR primers for analysis of *FUM14* expression level
FUM14-R	ACTCAGGAGCTGCGACTGATA	
FUM15-F	TATCGCCCTGGAAAAGCTTG	Quantitative real-time PCR primers for analysis of *FUM15* expression level
FUM15-R	TCTGTGGGGTCCATTCAATA	
FUM17-F	CTCACGATATCAGTGACCTTT	Quantitative real-time PCR primers for analysis of *FUM17* expression level
FUM17-R	AGCCATATGATGTTGAGGGT	
FUM18-F	TCCTCTTCCTGTTCCTGACGA	Quantitative real-time PCR primers for analysis of *FUM18* expression level
FUM18-R	TTGTAGTTGAGATTGCTGCCA	
FUM2-F	AAGTGCTCGGGGAGCGGGTT	Quantitative real-time PCR primers for analysis of *FUM2* expression level
FUM2-R	TCGGGGCATAACTCTATATCG	
FUM3-F	ACTGATTTCACCGAGGCCAA	Quantitative real-time PCR primers for analysis of *FUM3* expression level
FUM3-R	AGCGGACCGGAAGCTTCT	

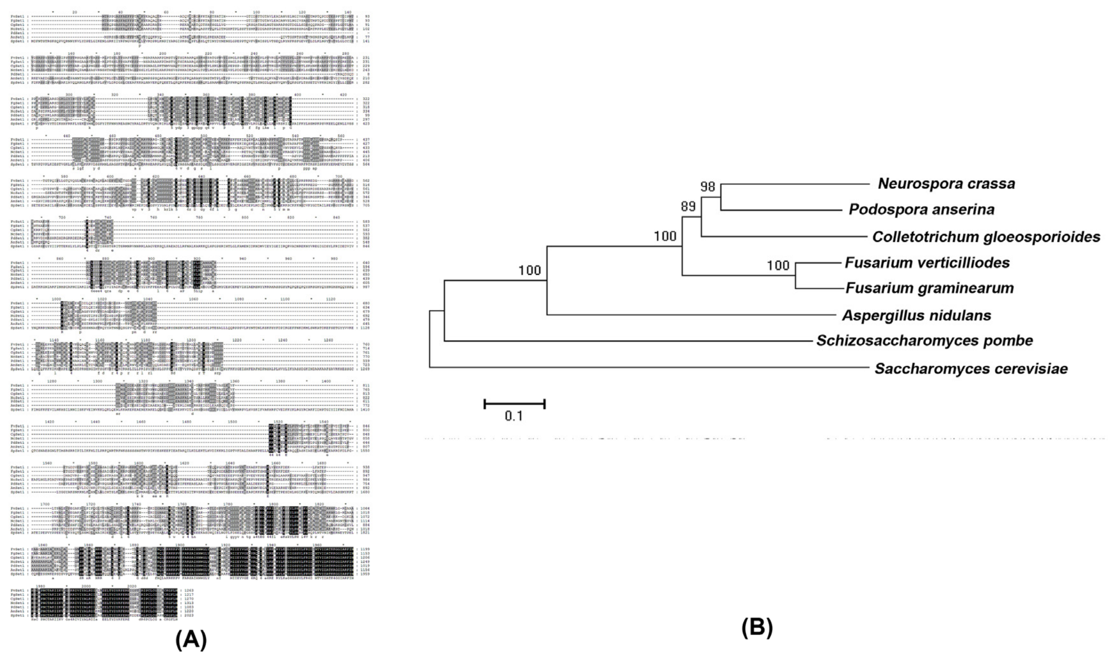

Figure S1. FvSet1 is homologous to those counterparts from yeasts and other filamentous fungi. (**A**) alignments of amino acid sequences of Set1 orthologs from *Fusarium verticillioides* (FvSet1), *Saccharomyces cerevisiae* (ScSet1), *Schizosaccharomyces pombe* (SpSet1), *Neurospora crassa* (NcSet1), *Podospora anserina* (PaSet1), *Colletotrichum gloeosporioides* (CgSet1), *Aspergillus nidulans*(AnSet1), and *Fusarium graminearum* (FgSet1). Boxshade program was used to highlight identical (black shading) or similar (grey shading) amino acids; (**B**) phylogenetic tree generated using the neighbour-joining method with Mega 4.1 software (http://www.megasoftware.net/) on the basis of the deduced amino acid sequences of FvSet1 from *F. verticillioides* strain 7600 and those from *S. cerevisiae*, *S. pombe*, *N. crassa*, *C. gloeosporioides*, *P. anserine*, *A. nidulans*, and *F. graminearum*. The bootstrap values are indicated on the phylogenetic tree.

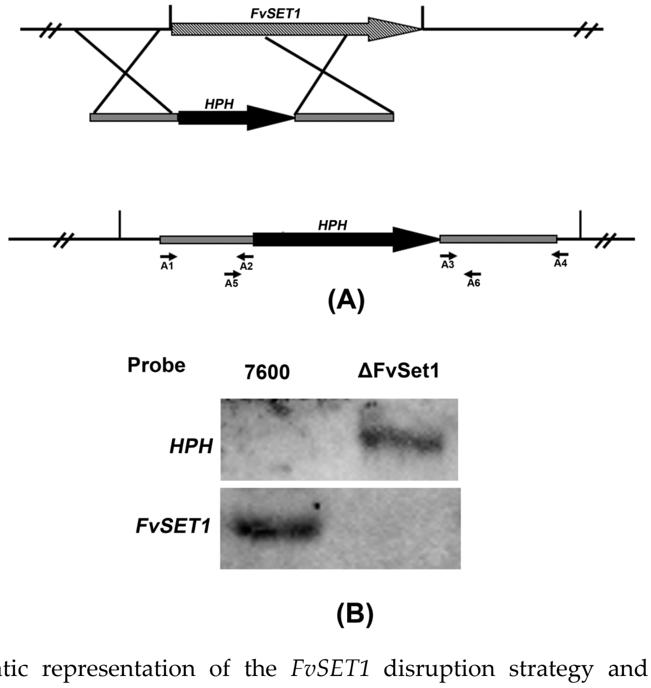

Figure S2. Schematic representation of the *FvSET1* disruption strategy and Southern blotting analyses of the deletion mutants. (**A**) Schematic diagram of the *FvSET1* gene, and gene replacement construct; (**B**) Southern blot analysis of *Dra* I-digested genomic DNA of the wild type 7600 and ΔFvSet1 mutant hybridized with the *FvSET1* gene and *HPH* probes, respectively.

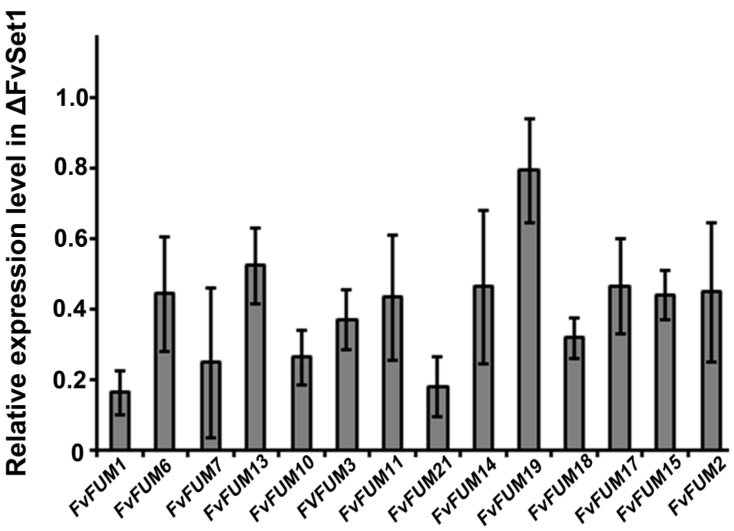

Figure S3. Relative expression of *FUM* genes in the ΔFvSet1. The relative expression levels of *FUM* genes in ΔFvSet1 are the relative amounts of mRNA of the gene in the wild-type progenitor. The expression level of the actin gene was used as an internal reference for each sample. Line bars in each column denote standard errors of three repeated experiments.
